# Plasma biomarkers of amyloid, tau & neuroinflammation in Alzheimer’s disease and corticobasal syndrome

**DOI:** 10.1007/s00406-025-02013-z

**Published:** 2025-05-02

**Authors:** Carolin Kurz, Laura Carli, Selim Üstün Gürsel, Isabelle Schrurs, Alexander Jethwa, Margherita Carboni, Tobias Bittner, Sayuri Hortsch, Daniel Keeser, Matthias Brendel, Lena Burow, Jan Haeckert, Carolin A. M. Koriath, Maia Tatò, Julia Utecht, Boris Papazov, Estrella Morenas-Rodriguez, Oliver Pogarell, Carla Palleis, Endy Weidinger, Sophia Stoecklein, Johannes Levin, Günter Höglinger, Boris-Stephan Rauchmann, Robert Perneczky

**Affiliations:** 1 Department of Psychiatry and Psychotherapy, LMU University Hospital, LMU Munich, Nußbaumstr. 7, 80336 Munich, Germany; 2 Roche Diagnostics GmbH, 82377 Penzberg, Germany; 3 Roche Diagnostics International Ltd, 6343 Rotkreuz, Switzerland; 4 F. Hoffmann-La Roche Ltd, 4070 Basel, Switzerland; 5 German Center for Neurodegenerative Diseases (DZNE), 81377 Munich, Germany; 6 Munich Cluster for Systems Neurology (SyNergy), 80336 Munich, Germany; 7 Department of Nuclear Medicine, LMU Hospital Munich, LMU Munich, 81377 Munich, Germany; 8 Department of Psychiatry, Psychotherapy and Psychosomatics, Medical Faculty, University of Augsburg, 86156 Augsburg, Germany; 9 Clinic for Psychiatry, Psychotherapy and Psychosomatics at the University of Augsburg, Augsburg, Germany; 10 Department of Radiology, LMU Hospital Munich, LMU Munich, 81377 Munich, Germany; 11 Institut de Recerca Hospital Sant Pau, 08041 Barcelona, Spain; 12 Department of Neurology, LMU Hospital Munich, LMU Munich, 81377 Munich, Germany; 13 Department of Neuroradiology, LMU Hospital Munich, LMU Munich, 81377 Munich, Germany; 14 Ageing Epidemiology (AGE) Research Unit, School of Public Health, Imperial College London, London, W6 8RP UK; 15 Sheffield Institute for Translational Neuroscience (SITraN), University of Sheffield, Sheffield, S10 2HQ UK

**Keywords:** Non-Alzheimer's disease dementia; beta, Beta-amyloid 1-40 (Aβ1-40), Beta-amyloid 1-42 (Aβ1-42), Phosphorylated tau (pTau), Neurofilament light chain (NfL), Glial fibrillary acidic protein (GFAP), Apolipoprotein E (ApoE4)

## Abstract

**Background:**

Blood-based biomarkers (BBBMs) could significantly facilitate the diagnosis of Alzheimer’s disease (AD) and non-AD dementia by providing less invasive alternatives to cerebrospinal fluid (CSF) and positron emission tomography (PET) imaging.

**Objective:**

This study investigated how well the BBBMs—amyloid-β (Aβ) 1-42/1-40 ratio, phosphorylated tau181 (pTau181), apolipoprotein E4 (ApoE4), glial fibrillary acidic protein (GFAP), and neurofilament light chain (NfL)—reflect thorough clinical work-up validated by PET and CSF biomarkers in participants with AD (n = 27), Aβ-negative CBS (n = 26), and agematched healthy controls (HC) (n = 17).

**Methods:**

Factor and correlation explored biomarker associations. Bayesian regression, backward selection regression, and ROC curve analysis were applied to identify optimal biomarker combinations and diagnostic cut-offs.

**Results:**

In AD cases, pTau181 and ApoE4 levels were elevated, and the Aβ1-42/1-40 ratio was reduced. ROC analysis showed high accuracy for pTau181, ApoE4 and Aβ1-42/1-40 in discriminating AD from HC, with a combination significantly improving performance. However, limited fold change, and high variability reduced the diagnostic applicability of Aβ1-42/1-40 ratio. Elevated NfL levels were the most reliable biomarker for CBS-Aβ(–) cases. GFAP showed limited discriminatory power due to overlapping levels, suggesting that it may not serve as a disease-specific biomarker but may be indicative of general neurodegeneration.

**Conclusions:**

This study highlights the diagnostic utility of pTau181, ApoE4 and the Aβ1-42/1-40 ratio for AD and NfL in the CBS-Aβ(–) cases and emphasizes the added value of combined biomarker models for group differentiation. Prospective studies will help validate these findings and refine clinical thresholds.

**Supplementary Information:**

The online version contains supplementary material available at 10.1007/s00406-025-02013-z.

## Introduction

Cerebrospinal fluid (CSF) biomarkers and neuroimaging, particularly targeting amyloid and tau pathology, have advanced the diagnosis of Alzheimer's disease (AD), particularly towards its early stages. However, these methods remain invasive, costly and largely inaccessible, highlighting the need for easy-to-use alternatives [1]. Blood-based biomarkers (BBBMs), including the Aβ1-42/1-40 ratio and phosphorylated tau181 (pTau181), have demonstrated significant diagnostic value in AD as indicators of underlying tau and amyloid pathology [2–6]. However, these biomarkers primarily target AD pathology and are less informative for other neurodegenerative conditions, such as corticobasal syndrome (CBS). Despite advances in AD diagnostics, there remains a significant unmet need for biomarkers that can reliably differentiate non-AD dementias and healthy controls from AD in routine clinical practice.

Indeed, CBS poses a unique diagnostic challenge, as it can mimic AD through overlapping symptoms like cognitive decline and apraxia [7]. CBS can arise from several non-AD pathologies, including corticobasal degeneration (CBD), progressive supranuclear palsy (PSP), or TAR DNA-binding protein 43 (TDP-43) proteinopathy, or can occur as a coexisting pathology [7–10]. Mixed pathologies, such as coexisting AD and non-AD pathology, are known to have negative synergistic effects on disease progression and prognosis, underscoring the necessity of biomarkers to disentangle these complexities and refine differential diagnosis [11, 12].

To address this gap, we investigated biomarkers beyond amyloid and tau that reflect complementary aspects of neurodegeneration: apolipoprotein E (ApoE4), glial fibrillary acidic protein (GFAP), and neurofilament light chain (NfL). ApoE4, the strongest genetic risk factor for late-onset AD, provides insights into genetic risk, aiding in the identification of individuals with elevated or reduced risk and complementing other biomarkers in stratifying amyloid pathology [13]. GFAP, a marker of astroglial activation, is valuable for identifying neuroinflammatory processes, while NfL is highly sensitive to neurodegeneration and shows promise in distinguishing AD from non-AD dementias like frontotemporal dementia and CBS [14–16].

This study aims to improve the diagnosis of CBS and other non-AD dementias using BBBMs, with a particular focus on discriminating between AD, CBS and healthy controls. A better understanding of these biomarkers will be a step towards more personalized and effective management of dementia, improving patient care and access to healthcare.

## Methods and materials

### Study design and participants

This study was designed as a prospective cohort study to evaluate the diagnostic value of Aβ1-42/1-40, pTau181, ApoE4, GFAP, and NfL. This study was conducted within the framework of the “Activity of Cerebral Networks, Amyloid, and Microglia Activity in Aging and Alzheimer’s Disease” (ActiGliA) study.

The study, initiated in 2017, was approved by the local ethics committee of LMU Munich (project numbers 17-755 and 17-569) and registered at clinicaltrials.gov (NCT06224920). Written informed consent was obtained from all participants in accordance with the Declaration of Helsinki. Recruitment took place between May 2018 and November 2021 through the outpatient clinics of the Departments of Psychiatry and Psychotherapy and Neurology (both at LMU Hospital Munich). The ability to give consent was systematically assessed [17]. Under the responsibility of specialists in neurology and/or psychiatry, participants underwent a comprehensive clinical evaluation, including examination of CSF, standardized neurological and psychiatric assessments, neuropsychological testing and brain imaging.

### Participants with AD dementia and MCI due to AD

Participants with probable AD dementia and MCI due to AD were categorized to capture the spectrum of AD progression. Both groups, along with healthy controls (HC), underwent comprehensive cognitive testing, including CERAD (Consortium to Establish a Registry for Alzheimer’s Disease), CDR (Clinical Dementia Rating), MMSE (Mini-Mental State Examination), ADAS-Cog (Alzheimer’s Disease Assessment Scale-Cognitive Subscale), and FCRST (Free and Cued Recall Selective Reminding Test). Probable AD dementia was defined by cognitive decline affecting daily living, confirmed by standardized tests and structured interviews with informants [18, 19]. MCI due to AD was identified by objective cognitive decline and preserved independence in daily living [19]. MMSE was used to estimate global cognition across all groups, with detailed results available upon request [20].

### Participants with corticobasal syndrome

Participants with CBS were diagnosed based on established criteria for presentations such as asymmetric parkinsonism, frontal-behavioral-spatial syndrome, and apraxia [7, 9, 21, 22]. CBS cases were classified as CBS-Aβ( +) or CBS-Aβ(–) based on the results of CSF Aβ1-42/1-40 ratios and/or amyloid PET findings [7, 8].

### Healthy controls (HC)

Healthy age-matched controls (HCs) were defined by the absence of AD pathology (normal amyloid PET or CSF Aβ1-42/1-40 ratio), structural brain abnormalities, and normal neurological, psychiatric, and neuropsychological evaluations, with scores within one standard deviation below the mean [23].

### Positron emission tomography (PET)

Participants were scanned with [18F]flutemetamol, an FDA and EMA approved amyloid PET tracer, at the Department of Nuclear Medicine, LMU Hospital [24]. Briefly, study participants were scanned on a Biograph 64 or a Siemens mCT PET/C scanner (Siemens, Erlangen, Germany), after a baseline CT scan, dynamic emission imaging was performed 0–60 min after injection of the radionuclide. PET data were reconstructed by recombining the baseline and the dynamic emission recordings. Standardized uptake value ratios (SUVr) of all 246 volumes of interest of the brainnetome atlas were extracted and used for data analysis.

### Analysis of cerebrospinal fluid

Immunoassays from Fujirebio^®^ (Gent, Belgium) (phosphoTau 181), IBL International (Hamburg, Germany) (total tau, Aβ1-40 and Aβ1-42) were used and their values were interpreted as indicative of AD according to the values established by the LMU laboratory: pTau181 > 61 pg/ml, Aβ1-42/1-40 ratio < 0.055 and total tau > 445 pg/ml [25].

### Definition of amyloid positivity

Amyloid positivity was defined either as a CSF Aβ1-42/1-40 ratio below 0.055 according to LMU Hospital laboratory procedures or by assessment of [18F]flutemetamol amyloid PET tracer retention [26, 27].

### Analysis of BBBM

Blood was collected and processed according to the standard procedures of the Munich Mental Health Biobank [28]. Samples from CBS-Aβ(–) and CBS-Aβ( +) cases underwent one freeze–thaw cycle prior to this analysis. The analysis of BBBM (Aβ1-42, Aβ1-40, pTau181, ApoE4, NfL, GFAP) was performed using a Cobas^®^ e601/e411 module/analyzer (Roche Diagnostics, Switzerland) based on the Elecsys electrochemiluminescence immunoassay technology (all Roche Diagnostics Internation Ltd, Rotkreuz, Switzerland). The Elecsys assays used in this study are part of the NeuroToolKit, a panel of exploratory robust prototype assays (Roche Diagnostics Internation Ltd, Rotkreuz, Switzerland). The analysis was performed in a single batch using a quantitative sandwich assay with a determination time of 18 min. Analyte-specific antibodies labelled with biotin or a ruthenium complex bind to streptavidin-coated microparticles, which are magnetically captured on an electrode. After washing with ProCell M solution, a voltage is applied to induce chemiluminescent emission, which is measured by a photomultiplier.

### *APOE4* ε4 genotyping

APOE4ε genotypes were determined using TaqMan SNP assays after automated DNA isolation from EDTA blood.

### Statistical analyses

Extreme outliers were removed using the IQR method and data were z standardized. Due to non-normal distributions, non-parametric tests (Kruskal–Wallis and pairwise Mann–Whitney *U* tests) were used, with adjustments for age, sex and FDR correction for multiple testing. Small sample size groups were excluded. Spearman and partial Spearman correlations (adjusted for age and sex) with FDR correction assessed clinical-biomarker relationships. Factor analysis with varimax rotation identified latent factors, and Bayesian regression addressed multicollinearity (via VIF) and selected significant biomarkers. ROC analysis (AUC, sensitivity, specificity, Youden index) determined optimal cut-offs, with cross-validation ensuring reliability. Combined models optimized AUC and biomarker contributions, quantified using posterior distributions and credible intervals. A significance level of p < 0.05 was used. All statistical tests and graphs were generated using R Studio version 2024 [29].

## Results

### Recruitment of participants

**Fig. 1:**
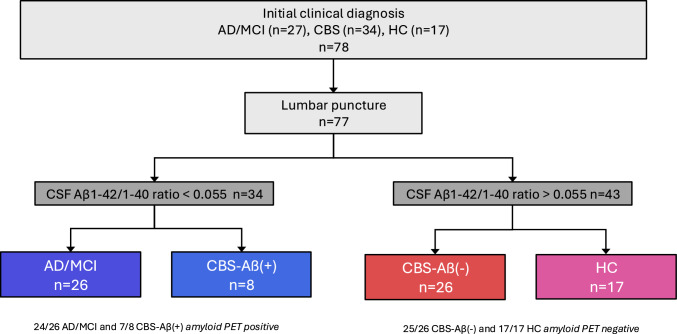
Recruitment and Group Classification. Of 138 participants, n = 31 withdrew their consent, n = 21 were excluded from this analysis due to missing data, n = 4 due to a diagnosis of small vessel disease (SVD), n = 4 due to indeterminate disease. In n = 2 cases amyloid PET was available but not CSF, in n = 5 cases CSF was available but not amyloid PET. One amyloid PET-negative case had a CSF amyloid ratio below 0.055, presented clinically with CBS and was classified as a CBS-Aβ( +) case. N = 78 participants underwent magnet resonance imaging. 76% were right-handed, 9% left-handed, 5% indifferent and 10% missing data

The final analysis included n = 77 participants with the following CSF- and amyloid-PET based clinical diagnoses (Fig. 1):

- Alzheimer´s disease (AD) (n = 26)
  o mild cognitive impairment due to AD (AD-MCI) (n = 14)
  o mild dementia due to AD (n = 12)
- Aβ-positive corticobasal syndrome (CBS-Aβ( +)) (n = 8)
- Aβ-negative corticobasal syndrome (CBS-Aβ(–)) (n = 26)
- Healthy controls (HC) (n = 17)

Participants with AD were predominantly in the early stages of the disease, as reflected by the subdivision into mild cognitive impairment (AD-MCI) and mild dementia, and the relatively preserved mean MMSE score (Table 1). PET imaging confirmed amyloid positivity in 24/26 AD/MCI cases, while 25/26 CBS-Aβ(–) and 17/17 HC were amyloid PET negative, supporting the classification of these participants (Fig. 1). Due to the Covid-19 pandemic, this study faced a high drop-out rate, which significantly limited the availability of follow-up data. Of the 78 participants, only 43 completed the 18-month follow-up, making it impossible to reliably analyze changes in BBBM levels over time.

**Table 1:** Demographic data (continuous variables) including age, duration of illness, Mini Mental Examination Status (MMSE) and education across diagnostic groups

Demographics—Continuous Variables								
Group	n	Mean	Median	SD	Pct25	Pct75	Shapiro (p value)	Kruskal (p value)
Age (years)								
AD	26	70.05	71.00	6.90	66.50	74.00		
CBS-Aβ( +)	8	74.89	75.00	9.10	72.00	83.00		
CBS-Aβ(–)	26	71.23	72.00	6.71	67.00	76.00		
HC	17	70.50	70.50	6.13	69.00	73.50		
All cases	77	71.93	72.00	7.08	67.00	76.50	0.065	0.329
Duration (months)								
AD	26	37.95	28.00	23.89	17.50	56.50		
CBS-Aβ( +)	8	33.89	25.00	20.60	17.00	51.00		
CBS-Aβ(–)	26	30.12	25.00	17.39	17.25	36.00		
HC	17	0.000	0.000	0.000	0.000	0.000		
All cases	77	32.90	27.50	19.42	17.00	45.00	0.000	0.556
Education (years)								
AD	26	15.11	14.00	3.40	13.00	17.00		
CBS-Aβ( +)	8	12.89	12.00	3.26	11.00	14.00		
CBS-Aβ(–)	26	13.15	13.00	2.75	11.00	14.00		
HC	17	14.90	14.00	3.21	12.25	17.50		
All cases	77	14.00	13.00	3.37	11.50	17.00	0.004	0.124
MMSE (0 to 30 points)								
AD	26	25.00	25.00	3.54	22.00	27.50		
CBS-Aβ( +)	8	19.56	22.00	8.63	14.00	26.00		
CBS-Aβ(–)	25	24.48	26.00	5.36	21.00	28.00		
HC	17	29.35	30.00	1.11	29.00	30.00		
All cases	78	25.49	27.00	4.94	24.00	29.00	0.000	0.000

### Participant characteristics and clinical data

Age, disease duration and education were not significantly different between the AD, CBS-Aβ(–) and HC groups, as indicated by the Kruskal–Wallis test (p > 0.05 each). Participants were generally in their early 70 s, with mean age ranging from 70.0 years (AD) to 74.9 years (CBS-Aβ( +)). Disease duration was longest in the AD group (mean = 37.9 months) and shortest in the CBS-Aβ(–) group (mean = 30.1 months). Educational attainment was relatively high in all groups, with a mean of 13.2 years (CBS-Aβ(–)) to 15.1 years (AD). However, MMSE scores were significantly higher in the HC group than in the AD and CBS-Aβ(–) groups (p < 0.001), reflecting preserved cognitive function in the healthy cohort (Tables 1 and 2).

**Table 2:** Post-hoc analysis for demographic differences between groups using Mann–Whitney tests

Post-hoc Analysis (Mann–Whitney-Test)				
	Age	Duration	Education	MMSE
AD vs. CBS-Aβ(–)	p > 0.05	p > 0.05	p > 0.05	p > 0.05
AD vs. HC	p > 0.05	p = 0.001	p > 0.05	p = 0.004
CBS-Aβ(–) vs. HC	p > 0.05	p = 0.001	p > 0.05	p = 0.004

Ordinal variables (pairwise Fisher´s-exact-Test)			
	Sex	Amyloid PET	ApoEε4 carrier status
AD vs. CBS-Aβ(–)	p > 0.05	p = 0.001	p = 0.001
AD vs. HC	p > 0.05	p = 0.001	p = 0.001
CBS-Aβ(–) vs. HC	p > 0.05	p > 0.05	p > 0.05

FDR-corrected, CBS-Aβ(+) excluded by minimum size filtering

### Categorial variables

Significant differences were observed in amyloid PET status (p = 0.001) and *ApoEε4* carrier status (p < 0.001), whereas the sex distribution was balanced between groups with no significant differences (p > 0.05). Pairwise comparisons showed that AD was significantly different from CBS-Aβ(–) and HC in both amyloid PET status and *ApoEε4* carrier status (p = 0.001 for each comparison) with the AD group largely consisting of *ApoEε4* carriers. No significant differences were found between CBS-Aβ(–) and HC in either parameter (p > 0.05) (Tables 3 and 4).

**Table 3:** Demographic data (categorical variables) including sex, amyloid PET results, and ApoEε4 carrier status across groups

Demographics—Categorial Variables								
		Sex		Amyloid PET		ApoEε4 carrier status		
		Female	Male	Positive	Negative	Non-carrier	Hemizygous	Homozygous
AD	26	17	10	24	0	6	15	6
CBS-Aβ( +)	8	4	4	7	1	4	2	2
CBS-Aβ(–)	26	15	11	0	25	15	4	4
HC	17	8	9	0	17	14	3	0
all cases	77	43	34	31	43	40	24	12
Overall Fisher´s exact test								
	p > 0.05			p = 0.001		p = 0.000		

p values FDR-corrected for multiple testing

**Table 4:** Post-hoc analysis for categorical demographic variables using Fisher’s exact test

Post-Hoc Analysis (pairwise Fisher´s-exact-Test)			
	Sex	Amyloid PET	ApoEε4 carrier status
AD vs. CBS-Aβ(–)	p > 0.05	p = 0.001	p = 0.001
AD vs. HC	p > 0.05	p = 0.001	p = 0.001
CBS-Aβ(–) vs. HC	p > 0.05	p > 0.05	p > 0.05

FDR-corrected, CBS-Aβ( +) excluded by minimum size filtering

### CBS-Aβ( +) group

The CBS-Aβ( +) group had a small sample size (n = 8), which limited the ability to perform statistical analyses. However, descriptive data suggest that participants in this group had a mean age of 74.9 years and a mean disease duration of 33.9 months, both slightly higher than in the other groups. Educational attainment in this group was slightly lower (mean = 12.9 years) than in the AD group (mean = 15.1 years), but comparable to the CBS-Aβ(–) group. MMSE scores (mean = 19.6 p.) indicated significant cognitive impairment in comparison to the CBS-Aβ(–) group (mean = 24.4 p.) (Tables 1 and 2). Amyloid PET positivity in the CBS-Aβ( +) group was observed in seven out of eight cases, with no clear pattern of ApoEε4 carrier status as the group included both carriers and non-carriers. Due to the small sample size, these findings should be interpreted with caution. In addition, the present approach cannot distinguish whether the CBS-Aβ( +) group represents cases of corticobasal syndrome with AD co-pathology or a variant of AD with a corticobasal syndrome phenotype.

### Blood-based biomarkers

Mean levels of pTau181 were highest in the AD group (1.333 pg/ml) and the CBS-Aβ( +) group (1.222 pg/ml), with significantly lower levels observed in the CBS-Aβ(–) and HC groups (p < 0.001, Kruskal–Wallis) (Table 5). The post-hoc analysis revealed highly significant differences in pTau181 levels between AD and CBS-Aβ(–) (p < 0.001) as well as AD and HC (p = 0.000), with a moderate significance observed between CBS-Aβ(–) and HC (p = 0.004) (Table 6).

**Table 5:** Blood-based biomarker levels for pTau181, Aβ1-42/1-40, ApoE4, GFAP, and NFL across groups (FDR-adjusted p-values)

BBBM (p values corrected for multiple testing)								
Group	n	Mean	Median	SD	Pct25	Pct75	Shapiro (p value)	Kruskal (p value)
pTau181 (pg/ml)								
AD	25	1.333	1.250	0.604	0.842	1.480		
CBS-Aβ( +)	8	1.222	1.210	0.465	0.891	1.605		
CBS-Aβ(–)	21	0.715	0.728	0.148	0.627	0.823		
HC	17	0.735	0.678	0.254	0.592	0.807		
All cases	71	1.017	0.860	0.526	0.689	1.190	0.000	0.000
Aβ1-42/1-40 ratio								
AD	25	0.118	0.117	0.007	0.113	0.122		
CBS-Aβ( +)	8	0.112	0.111	0.007	0.109	0.116		
CBS-Aβ(–)	21	0.129	0.132	0.013	0.127	0.135		
HC	17	0.135	0.133	0.019	0.129	0.141		
All cases	71	0.124	0.123	0.016	0.114	0.133	0.000	0.000
ApoE4 (UG/ml)								
AD	25	9.920	9.780	7.759	7.330	11.620		
CBS-Aβ( +)	8	2.530	0.000	3.503	0.000	6.348		
CBS-Aβ(–)	21	2.342	0.000	5.145	0.000	0.000		
HC	17	1.578	0.000	3.525	0.000	0.000		
All cases	71	4.006	0.000	7.038	0.000	7.263	0.000	0.000
GFAP (ng/ml)								
AD	25	0.147	0.134	0.066	0.099	0.189		
CBS-Aβ( +)	8	0.146	0.165	0.053	0.139	0.179		
CBS-Aβ(–)	21	0.105	0.099	0.049	0.071	0.143		
HC	17	0.092	0.067	0.068	0.060	0.089		
All cases	71	0.124	0.114	0.069	0.069	0.161	0.002	0.003
NfL (pg/ml)								
AD	25	2.549	2.540	0.884	1.920	3.160		
CBS-Aβ( +)	8	3.598	3.345	1.197	2.685	4.685		
CBS-Aβ(–)	21	4.867	4.570	2.790	2.580	5.770		
HC	17	2.069	1.900	0.899	1.390	2.620		
All cases	71	3.457	2.720	2.387	1.930	4.080	0.000	0.002

**Table 6:** Post-hoc analysis of blood-based biomarker differences between groups using Mann–Whitney tests

Post-hoc Analysis (Mann–Whitney-Test)					
	pTau181 (pg/ml)	Aβ1-42/1-40 ratio	ApoE (UG/ml)	GFAP (ng/ml)	NfL (pg/ml)
AD vs. CBS-Aβ(–)	p = 0.000	p = 0.002	p = 0.003	p > 0.05	p = 0.002
AD vs. HC	p = 0.000	p = 0.002	p = 0.002	p = 0.003	p > 0.05
CBS-Aβ(–) vs. HC	p = 0.004	p > 0.05	p > 0.05	p > 0.05	p = 0.001

Adjusted for age and sex, FDR-corrected, CBS-Aβ( +) excluded by minimum size filtering

Similarly, the Aβ1-42/1-40 ratio was lowest in the AD (0.118) and the CBS-Aβ( +) groups (0.112), reflecting pathological changes typical of AD. In contrast, higher amyloid ratios were observed in the HC and CBS-Aβ(–) groups (0.135 and 0.129, respectively), with significant group differences detected (p = 0.000). For the Aβ1-42/1-40 ratio, highly significant differences were found between AD and CBS-Aβ(–) (p = 0.002) and AD and HC (p = 0.002), whereas no significant differences were observed between CBS-Aβ(–) and HC (p > 0.05).

**Fig. 2:**
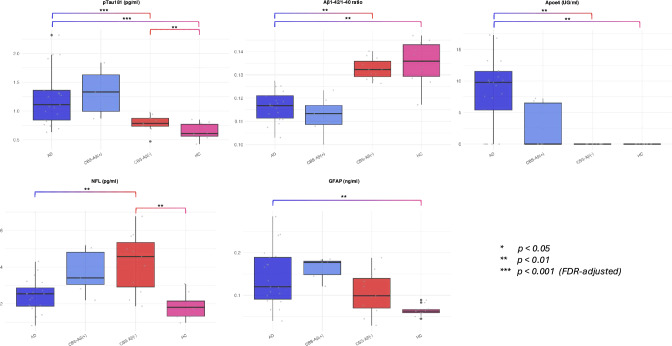
Blood-based Biomarkers—Raw Data and Group Differences. Blood-based biomarkers showing raw data and group differences for pTau181, Aβ1-42/1-40 ratio, ApoE4, NFL, and GFAP. Statistical significance: *p < 0.05, **p < 0.01, ***p < 0.001 (FDR-adjusted)

ApoE4 levels were highest in the AD group (9.920 UG/ml) and significantly lower in the HC group (1.578 UG/ml, p < 0.001). Intermediate levels were observed in the CBS-Aβ(–) group (2.342 UG/ml), while descriptive data for CBS-Aβ( +) indicated lower ApoE4 levels (2.530 UG/ml) like those of CBS-Aβ(–). In terms of ApoE4 levels, significant differences were detected between AD and CBS-Aβ(–) (p = 0.003) and AD and HC (p = 0.002), but CBS-Aβ(–) and HC showed no significant differences (p > 0.05). However, as shown in Table 3, a markedly higher proportion of *ApoEε4* carriers was found in the AD group compared to CBS-Aβ( +) and CBS-Aβ(–), where the number of *ApoEε4* carriers was notably lower. In fact, there were no ApoE4 carriers in the HC group. This suggests that rather than interpreting ApoE4 as a continuous biomarker, a dichotomous classification (*ApoEε4* carrier vs. non-carrier) may be more appropriate for understanding its role in disease risk and biomarker distribution. The boxplot in Fig. 2 further illustrates these differences, emphasizing the categorical nature of *ApoEε4* status.

GFAP levels were highest in the AD and CBS-Aβ( +) groups (0.147 and 0.146 ng/ml, respectively) and lowest in the HC group (0.092 ng/ml), while intermediate GFAP levels were observed in the CBS-Aβ(–) group (0.105 ng/ml). GFAP levels showed no significant differences between AD and CBS-Aβ(–) (p > 0.05) or CBS-Aβ(–) and HC (p > 0.05), but a significant result was observed when comparing AD and HC (p = 0.003).

Finally, NfL levels were highest in the CBS-Aβ(–) group (4.867 pg/ml) and lowest in the HC group (2.069 pg/ml), with significant group differences observed (p < 0.002). Descriptive data for the CBS-Aβ( +) group showed intermediate NfL levels (3.598 pg/ml). Finally, NfL levels exhibited highly significant differences between AD and CBS-Aβ(–) (p = 0.002) as well as CBS-Aβ(–) and HC (p = 0.001), while no significant differences were observed between AD and HC (p > 0.05).

These results highlight distinct biomarker profiles among the AD, CBS-Aβ(–) and HC groups, while the CBS-Aβ( +) group shows a biomarker pattern like AD cases, although statistical analysis was precluded by limited sample size (Fig. 2 and Tables 5 + 6).

### Cerebrospinal fluid markers

It is worth acknowledging that compared to their CSF counterpart, plasma amyloid ratios showed narrow interquartile ranges (e.g. AD 0.113–0.122, HC 0.129–0.141), small standard deviations (e.g. AD 0.007, HC 0.019) and substantial overlap in IQRs, e.g. between HC and CBS-Aβ(–) (0.127–0.135), which may limit their diagnostic utility by reducing the ability to clearly differentiate between groups. In contrast, CSF biomarkers showed wider interquartile ranges (e.g. AD 0.029–0.043, HC 0.068–0.084) with less overlap between groups, allowing clearer differentiation, especially between AD and HC.

When comparing groups, the data for CSF biomarkers (Aβ1-42/1-40 ratio, pTau181, tTau and sTREM2) showed significant differences between groups for most biomarkers except sTREM2 (p > 0.05).

The CSF Aβ1-42/1-40 ratio was significantly lower in the AD and CBS-Aβ( +) groups (mean 0.036 and 0.040, respectively) compared to the HC and CBS-Aβ(–) groups (0.078 and 0.080, respectively; p < 0.001). For pTau181 levels, the AD group revealed the highest concentrations (mean 87.268 pg/ml), followed by the CBS-Aβ( +) group (70.116 pg/ml). CBS-Aβ(–) and HC had significantly lower levels (49.311 and 49.469 pg/ml, respectively; p < 0.001). Post-hoc analysis showed that AD was significantly different from both HC and CBS-Aβ(–), while descriptive data for CBS-Aβ( +) indicated biomarker levels like those of AD. This supports the hypothesis of a shared pathology between AD and CBS-Aβ( +).

Similarly, tTau levels were elevated in AD (490.913 pg/ml) and CBS-Aβ( +) (362.868 pg/ml) compared to HC and CBS-Aβ(–) (223.588 and 261.885 pg/ml, respectively; p < 0.001). However, no obvious differences in tTau were observed between CBS-Aβ( +) and CBS-Aβ(–), suggesting some overlap in tau pathology within CBS subtypes. Due to the limited sample size, the CBS-Aβ( +) group was excluded from statistical comparisons according to the minimum group size filtering approach.

**Fig. 3:**
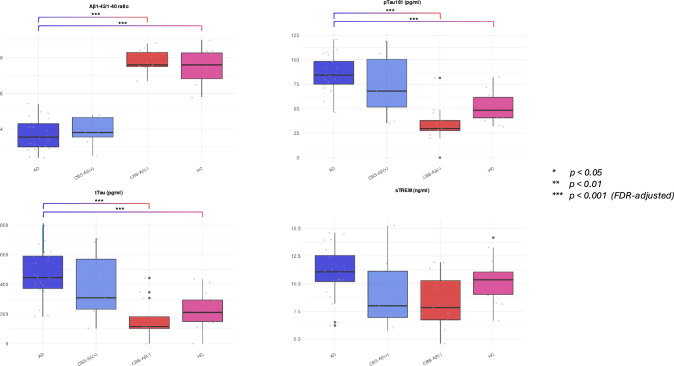
CSF Markers—Raw Data and Group Differences. CSF markers illustrating raw data and group differences for Aβ1-42/1-40 ratio, pTau181, tTau, and sTREM. Statistical significance: *p < 0.05, **p < 0.01, ***p < 0.001 (FDR-adjusted)

In contrast, sTREM2 levels were not significantly different between groups (p > 0,05), with similar levels observed in AD (11.305 ng/ml), CBS-Aβ( +) (9.316 ng/ml), CBS-Aβ(–) (9.930 ng/ml) and HC (10.477 ng/ml). These results suggest that sTREM2 had no discriminatory value in distinguishing between these disease groups (Fig. 3 and Tables 7 and 8).

**Table 7:** CSF biomarker levels (pTau181, Aβ1-42/1-40, tTau, sTREM2) across diagnostic groups with FDR-adjusted p-values.”

CSF markers baseline (p-values corrected for multiple testing)								
Group	n	Mean	Median	SD	Pct25	Pct75	Shapiro (p value)	Kruskal (p value)
pTau 181 (pg/ml)								
AD	26	87.268	84.375	19.759	75.030	98.238		
CBS-Aβ( +)	8	70.116	67.365	33.587	36.228	97.510		
CBS-Aβ(–)	26	49.311	36.655	39.707	28.705	50.818		
HC	17	49.469	50.190	19.380	40.650	58.780		
All cases	77	64.258	58.780	33.300	37.670	82.140	0.004	0.000
Aβ1-42/1-40 ratio								
AD	26	0.036	0.033	0.009	0.029	0.043		
CBS-Aβ( +)	8	0.040	0.040	0.008	0.037	0.046		
CBS-Aβ(–)	26	0.080	0.077	0.012	0.074	0.085		
HC	17	0.076	0.077	0.010	0.068	0.084		
All cases	77	0.060	0.066	0.023	0.038	0.079	0.001	0.000
tTau (pg/ml)								
AD	26	490.913	445.960	192.764	372.125	603.080		
CBS-Aβ( +)	8	362.868	279.135	230.730	206.253	512.528		
CBS-Aβ(–)	26	261.885	172.755	207.780	116.730	329.505		
HC	17	223.588	221.970	125.145	146.320	300.690		
All cases	77	344.766	315.485	218.064	175.955	445.653	0.002	0.000
sTREM2 (ng/ml)								
AD	26	11.305	11.075	3.857	9.510	12.950		
CBS-Aβ( +)	7	9.316	8.000	3.410	6.955	11.135		
CBS-Aβ(–)	26	9.930	9.885	3.126	7.360	11.910		
HC	17	10.477	10.355	3.079	8.775	11.365		
All cases	77	10.743	10.455	3.544	8.178	12.493	0.181	0.374

**Table 8:** Post-hoc analysis for CSF biomarkers between groups using Mann–Whitney tests

Post-hoc Analysis (Mann–Whitney-Test)				
	Aβ1-42/1-40 ratio	pTau181 (pg/ml)	tTau (pg/ml)	sTREM (ng/ml)
AD vs. CBS-Aβ(–)	p = 0.000	p = 0.000	p = 0.000	p > 0.05
AD vs. HC	p = 0.000	p = 0.000	p = 0.000	p > 0.05
CBS-Aβ(–) vs. HC	p > 0.05	p > 0.05	p > 0.05	p > 0.05

Adjusted for age and sex, FDR-corrected, CBS-Aβ( +) excluded by minimum size filtering

### Factor analysis

Two main components were identified by factor analysis of the BBBM, explaining a total of 60.966% of the variance, an excellent result for factor analysis. The communalities for pTau181 (0.676) and NfL (0.743) suggested that these variables were well explained by the model, whereas the Aβ1-42/1-40 ratio (0.286) had a weaker representation. Component 1 was strongly associated with pTau181 (0.822) and GFAP (0.770), suggesting that these variables contribute most to this factor, possibly representing a neurodegenerative and inflammatory process. Component 2 was strongly associated with ApoE4 (– 0.703) and NfL (0.817), indicating that it may capture another biological mechanism, such as axonal degeneration or lipid metabolism. The Aβ1-42/1-40 ratio had moderate loadings on both components (– 0.491 and 0.213), meaning that it was not strongly associated with either component, but still had relevance.

Factor analysis of the CSF biomarkers revealed that two main components explained 85.2% of the variance in the data, indicating that these markers provide a robust and highly structured data set for differentiation. High communalities for CSF pTau181 (0.861) and CSF Aβ1-42/1-40 ratio (0.748) suggested that these markers explained a substantial proportion of the variance and were critical to the data structure. Component 1 was associated with pTau181 CSF (0.860), tTau CSF (0.850) and sTREM2 (0.953), reflecting their contribution to neurodegenerative and inflammatory processes. Although the absolute levels of sTREM2 may not be significantly different between groups, the high commonalities indicate that it is well aligned with other biomarkers involved in inflammation (e.g. tTau or pTau181), contributing to the latent neurodegenerative factor. Component 2 was predominantly characterized by the Aβ1-42/1-40 CSF ratio (– 0.855), consistent with its relevance in amyloid pathology. The high loadings for these markers suggest that they represent the most influential variables in differentiating the data structure (Table 9).

**Table 9:** Factor analysis of blood and CSF biomarkers showing explained variance and factor loadings

Factor Analysis BBBM										
Variance explained				Communalities			Rotated component matrix		Measure	Bartlett's Test
Component	Eigenvalue	% Variance	Cumulative variance	Variable	Initial	Extraction	Component_1	Component_2	Kaiser–Meyer–Olkin	p value
1	1.786	35.729	35.729	Aß1-42/1-40 ratio	1	0.286	– 0.491	0.213	0.555	< 0.001
2	1.262	25.236	60.966	ApoE4 (UG/ml)	1	0.695	– 0.448	– 0.703		
3	0.889	17.775	78.74	GFAP (ng/ml)	1	0.648	0.770	0.235		
4	0.598	11.95	90.69	NFL (pg/ml)	1	0.743	0.275	0.817		
5	0.465	9.31	100	pTau181 (pg/ml)	1	0.676	0.822	0.007		
CSF markers										
1	2.507	0.552	0.552	pTau181 CSF (pg/ml)	1	0.861	0.860	0.348	0.683	< 0.001
2	0.899	0.300	0.852	Aß1-42/1-40 ratio	1	0.748	– 0.855	0.134		
3	0.475	0.552	0.552	tTau181 (pg/ml)	1	0.875	0.850	0.390		
4	0.118	0.300	0.852	sTREM2 (ng/ml)	1	0.922	0.116	0.953		

### Correlation of BBBM and CSF biomarkers

**Fig. 4:**
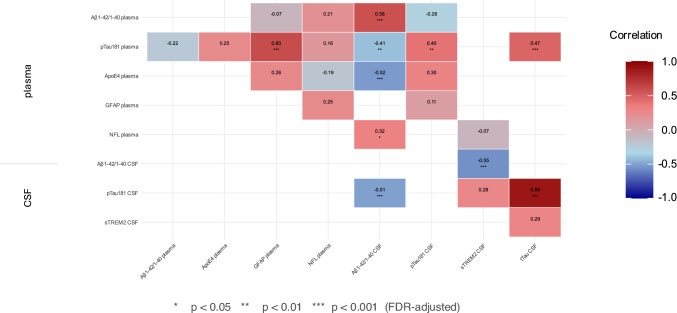
Correlation of Blood-based Biomarkers and CSF Markers. Correlation analysis of blood-based biomarkers with CSF markers. Statistical significance: *p < 0.05, **p < 0.01, ***p < 0.001 (FDR-adjusted)

Correlation analyses, adjusted for age and sex and corrected for multiple testing using FDR, revealed significant relationships between plasma and CSF biomarkers, highlighting the interplay between amyloid deposition, tau pathology and neurodegeneration (Fig. 4).

Plasma and CSF pTau181 levels were moderately correlated (r = 0.398; p = 0.005), suggesting that plasma pTau181 may serve as a surrogate marker of central tau pathology. Plasma GFAP correlated strongly with plasma pTau181 (r = 0.601; p = 0.000), linking glial activation with tau processes. The CSF Aβ1-42/1-40 ratio was strongly negatively correlated with CSF pTau181 (r = – 0.505; p = 0.000) and CSF tTau (r = – 0.553; p = 0.000), reflecting the inverse relationship between amyloid deposition and tau accumulation. Plasma Aβ1-42/1-40 correlated weakly with plasma ApoE4 (r = – 0.322; p = 0.033) and CSF pTau181 (r = – 0.284; p = 0.0057) and moderately with its CSF counterpart (r = 0.578; p = 0.000), suggesting a partial overlap between peripheral and central amyloid markers. Given the strong association between *ApoEε4* carrier status and AD pathology, it is important to consider whether these correlations are driven by the presence of *ApoEε4* carriers rather than absolute protein levels.

CSF pTau181 and tTau showed a very strong correlation (r = 0.885; p = 0.000), suggesting their complementary reflection of tau-related neurodegeneration. Plasma NfL correlated moderately with CSF Aβ1-42/1-40 (r = 0.316; p = 0.035), reflecting the opposing pathological signatures of axonal damage and amyloid burden in the different groups. Finally, plasma GFAP showed a moderate correlation with plasma NfL, which was initially significant. However, this significance disappeared after adjustment for age, sex and multiple testing corrections (r = 0.253; p = 0.090).

**Fig. 5:**
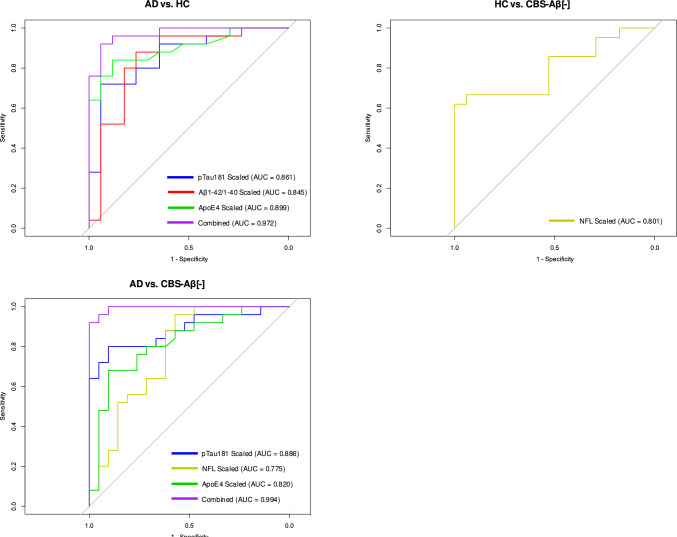
Blood biomarker-based Prediction versus CSF/PET-based Diagnosis. Comparison of blood biomarker-based predictions versus CSF/PET-based diagnoses. AUC values indicate the predictive power of scaled biomarkers and combined models

The variance inflation factor (VIF) values for the analyzed biomarkers were below the commonly accepted threshold of 10 (ranging from 1.30 to 5.72), indicating no evidence of severe multicollinearity in the data. Taken together, these findings underscored the potential of plasma biomarkers to reflect central neurodegenerative processes, while highlighting important differences in peripheral and central biomarker dynamics or metabolism (Fig. 5 and Table 1, Supplementary Appendix).

### Diagnostic performance and utility of biomarkers

The diagnostic performance of the biomarkers varied significantly between disease groups, highlighting their strengths and limitations. Regression with backward selection identified pTau181, ApoE4 and NfL as important predictors of group differentiation, while the Aβ1-42/1-40 ratio presented specific limitations. Age was also significant in some comparisons, reflecting its role as a confounding factor. The inclusion of combined biomarker models further improved discrimination, as confirmed by ROC analyses (Table 10 and Fig. 5).

**Table 10:** Predictors of disease group differentiation based on biomarker regression analysis

Predictors of Disease Group Differentiation (Regression with backward selection)								
Group	Term	Estimate	Std.error	Statistic	P value	Conf.low	Conf.high	Adjusted p value (FDR)
AD	Intercept	0.000	7.048	– 2.512	0.012	0.000	0.007	0.007
	pTau181 (pg/ml)	5.81 E + 3	2.059	4.210	0.000	183.739	6.852 E + 5	0.000
	Aß1-42/1-40 ratio	0.000	23.364	– 1.310	0.190	0.000	4.702 E + 4	0.166
	GFAP (ng/ml)	0.019	6.432	– 0.616	0.538	0.000	2.487 E + 3	0.538
	NFL (pg/ml)	0.085	0.715	– 3.447	0.001	0.016	0.279	0.000
	ApoE4 (UG/ml)	1.381	0.088	3.654	0.000	1.185	1.690	0.000
	Sex	0.355	0.748	– 1.384	0.166	0.074	1.460	0.125
	Age	1.278	0.082	2.984	0.003	1.110	1.540	0.001
CBS-Aβ(–)	Intercept	6.957 E + 3	4.294	2.060	0.039	2.120	5.537 E + 7	0.059
	pTau181 (pg/ml)	0.002	1.467	– 4.396	0.000	0.000	0.018	0.000
	Aß1-42/1-40 ratio	0.002	15.335	– 0.411	0.681	0.000	8.169 E + 9	0.681
	NFL (pg/ml)	5.657	0.380	4.555	0.000	2.968	13.328	0.000
	Sex	0.693	0.616	– 0.595	0.552	0.200	2.318	0.662
	Age	0.886	0.050	– 2.407	0.016	0.797	0.973	0.032
HC	Intercept	0.000	4.707	– 2.530	0.011	0.000	0.034	0.030
	pTau181 (pg/ml)	0.677	0.995	– 0.393	0.695	0.075	3.719	0.695
	Aß1-42/1-40 ratio	3.368 E + 41	24.199	3.951	0.000	5,222 E + 5	2.037 E + 11	0.001
	GFAP (ng/ml)	0.007	4.821	– 1.020	0.308	0.000	82.816	0.410
	NFL (pg/ml)	0.317	0.364	– 3.157	0.002	0.141	0.584	0.006
	ApoE4 (UG/ml)	0.889	0.066	– 1.770	0.077	0.768	1.002	0.133
	Sex	2.753	0.585	1.732	0.083	0.898	9.135	0.133
	Age	1.032	0.042	0.759	0.448	0.951	1.124	0.512

Adjusted for age and sex, FDR-corrected, CBS-Aβ( +) excluded by minimum size filtering

### AD vs. HC

For discriminating AD from HC, the combined model of pTau181, ApoE4 and Aβ1-42/1-40 achieved the highest diagnostic accuracy (AUC = 0.972) with a sensitivity of 88.0% and specificity of 94.0%. On its own, ApoE4 (AUC = 0.832) showed excellent specificity (82.4%) at a cut-off of > 1.759 for AD cases. However, given the clear distinction in ApoE4 presence between AD and non-AD groups, a dichotomous classification (*ApoEε4* carrier vs. non-carrier) may provide a more robust and clinically interpretable measure.

It was the most reliable biomarker in this comparison, reflecting its association with AD pathology. For pTau181 (AUC = 0.876) a sensitivity of 70% and specificity of 94% was observed at a cut-off of > 1.349, highlighting its utility as a core biomarker. Regression analysis reinforced the importance of these biomarkers, with pTau181 showing the strongest effect size (estimate = 5.813, p < 0.001), followed by ApoE4 (estimate = 1.381, p < 0.001). Age also contributed significantly to group differentiation (Estimate = 1.278, p = 0.001), highlighting the importance of demographic factors in AD diagnosis.

High Aβ1-42/1-40 ratios were indicative of healthy controls (HC), as shown by regression analysis (Table 10; estimate = 3.368 E + 41, p = 0.001) and diagnostic performance metrics (Table 11; AUC = 0.836, sensitivity = 88.9%, specificity = 76.5%, Youden index = 0.645). Cut-off values of < 0.132 discriminated AD cases very well from HC (Fig. 2). However, modest differences between AD (mean 0.118) and HC (mean 0.135) with a ~ 17% reduction in AD and a small dynamic range (~ 0.833-fold change) reduced its reliability for differentiation. The high variability, wide confidence intervals and standard deviations, with lower confidence limits approaching zero, further indicated limited discriminatory power and substantial group overlap (Tables 10 and 11).

**Table 11:** Diagnostic performance of biomarkers for disease differentiation using Bayesian regression

Diagnostic Performance of Biomarkers							
Group	Biomarker	AUC	Sensitivity	Specificity	Youden	Standardized cut-off	Original cut-off
AD vs. HC	pTau181	0.876	0.704	0.941	0.645	0.675	1.349
	Aβ1-42/1-40	0.836	0.889	0.765	0.654	0.518	0.132
	ApoE4	0.832	0.778	0.824	0.601	0.478	1.759
AD vs. CBS-Aβ(–)	pTau181	0.892	0.704	0.963	0.665	0.665	1.394
	NfL	0.738	0.960	0.571	0.531	0.318	4.189
	ApoE4	0.819	0.741	0.923	0.664	0.613	2.179
CBS-Aβ(–) vs. HC	NfL	0.786	0.577	0.941	0.518	0.717	1.997

Bayesian regression adjusted for age and sex

Initial model included pTau181, Aβ1-42/1-40, ApoE4, NfL and GFAP, non-significant predictive contribution not shown

### AD vs. CBS-Aβ(–)

Discrimination between AD and CBS-Aβ(–) was most effective using pTau181, ApoE4 and NfL, with the combined model achieving an AUC of 0.994, coming close to the performance of an intensive clinical work-up. Individually, pTau181 (AUC = 0.892) showed sensitivity (70%) and specificity (96%) at a cut-off of > 1.394. Its strong performance highlights its role as a key biomarker for AD. ApoE4 (AUC = 0.819) showed a moderate sensitivity (74.1%) but a high specificity of 92.3% at a cut-off of > 2.179. NfL (AUC = 0.738) had a sensitivity of 96.0% but limited specificity (57.1%) at a cut-off of > 4.189 for CBS-Aβ(–) cases. Backward regression identified NfL (estimate = 5.657, p < 0.001) as the most influential biomarker, with age also contributed significantly (estimate = 0.886, p = 0.032). The strong AUC values highlight the complementary role of these markers in distinguishing AD from CBS-Aβ(–).

### HC vs. CBS-Aβ(–)

Differentiating HC from CBS-Aβ(–) was more challenging, with NfL emerging as the most effective biomarker: NfL achieved an AUC of 0.786, with high specificity (94.1%) and low sensitivity (57.7%) at a cut-off of > 1.997 for CBS-Aβ(–) cases. Other biomarkers, including pTau181, Aβ1-42/1-40 and ApoE4, showed minimal discriminatory ability. Regression analysis supported the central role of high Aß1-42/1-40 ratio (estimate = 3.368 E + 41, p = 0.001) for the identification of HC and NfL (estimate = 0.317, p = 0.006) for CBS-Aβ(–) cases. The sensitivity issue with NfL in diagnosing CBS-Aβ(–) was likely due to the fact that only very high NfL levels (IQR: 2.580–5.770, Table 5) strongly indicated CBS-Aβ(–), while lower levels overlapped with other groups such as HC (IQR: 1.390–2.620) and AD (IQR: 1.920–3.160), highlighting the need for additional biomarkers or alternative diagnostic approaches (Fig. 5 and Tables 10, 11, 12).

**Table 12:** Logistic regression improvement test comparing individual and combined biomarkers for predictive power

Logistic Regression Improvement Test (K1 Logistic Regression)					
AD vs. HC					
Comparison	Residual Df	Residual Dev	Df	Deviance	P-value
AD vs. CBS-Aβ(–)					
pTau vs. Base	40	38.892	1	17.8	< 0.001
Aβ vs. Base	40	39.425	1	17.266	< 0.001
ApoE4 vs. Base	40	31.863	1	24.828	< 0.001
pTau + Aβ vs. Base	39	28.54	2	28.151	< 0.001
pTau + ApoE4 vs. Base	39	20.111	2	36.58	< 0.001
HC vs. CBS-Aβ(–)					
pTau vs. Base	44	36	1	27	< 0.001
NFL vs. Base	44	47	1	16	< 0.001
ApoE4 vs. Base	44	48	1	16	< 0.001
Combined vs. pTau	42	7	2	29	< 0.001
Combined vs. NFL	42	7	2	40	< 0.001
Combined vs. ApoE4	42	7	2	41	< 0.001

Not applicable, only 1 biomarker in the model

Regression analyses highlighted the critical role of combined biomarker models, especially for comparisons involving AD cases. Individually, ApoE4, Aβ1-42/1-40 and pTau181 were the strongest performers for AD diagnosis, while NfL played a central role in CBS-Aβ(–) differentiation. For HC, the limited specificity of current biomarkers underlines the need for further research to refine diagnostic tools and improve differentiation from pathological cases.

## Discussion

In this study, we gained valuable insights into the diagnostic utility of blood-based biomarkers (BBBMs) in Alzheimer's disease (AD) and corticobasal syndrome (CBS).

The excellent discriminatory power of pTau181 in distinguishing AD from HC is consistent with previous studies and suggests that plasma pTau181 represents a reliable plasma surrogate for AD pathology [6, 14, 30, 31]. While pTau217 has shown promising results in early-stage detection and stronger correlations with both amyloid and tau PET imaging, pTau181 remains a reliable and extensively validated marker, particularly in moderate to advanced disease stages. [4] Additionally, pTau181 offers distinct advantages, including greater robustness to confounding factors such as renal function [32]. Further longitudinal studies are necessary to fully elucidate the comparative trajectories of pTau181 and pTau217 across all stages of AD [33].

High Aβ1-42/Aβ1-40 ratio was a significant predictor of HC in this study, plasma levels correlated with CSF Aβ1-42/1-40, which is consistent with previous studies. The use of Aβ1-42/1-40 presented significant limitations [10, 14]: In particular, the ratio had a large confidence interval and the small dynamic range (fold change) between amyloid-positive and amyloid-negative cases, as reported in previous studies underlined the limited robustness of this biomarker [5, 34]. These results suggest that although the plasma Aβ1-42/1-40 ratio may theoretically contribute to disease modelling, its narrow fold change increases the risk that even minor fluctuations—such as those induced by metabolic influences—could distort results and compromise the biomarker's interpretability [35–37]. An additional challenge were the extremely low biomarker levels in plasma, typically in the picogram range, which may increase susceptibility to both analytical and pre-analytical variability and highlight the need for strict standardization of protocols [34, 38, 39].

In the present analysis, ApoE4 levels were elevated in AD cases but there are contradicting reports of reduced ApoE4 levels in AD [40]. As plasma ApoE4 levels remain relatively underexplored in the literature, our study offers a systematic contribution to this area of research. The increase in ApoE4 production may reflect a response to amyloid deposition, inflammatory processes, or lipid transport activity [13, 40–43]. Alternatively, it could be primarily related to *ApoEε4* carrier status, rather than representing a continuous biomarker response. This distinction underscores the need for further investigation into whether a dichotomous classification (*ApoEε4* carrier vs. non-carrier) provides a more informative clinical and biological interpretation. ApoE4 improved AD classification over and above the contributions of pTau181 and Aβ1-42/40, providing an independent diagnosis. Factor analysis grouped ApoE4 with NfL on a distinct component, suggesting a link to neurodegeneration and axonal damage. This indicated that ApoE4 captures complementary pathological processes beyond tau and amyloid, such as neuroinflammation or neuronal repair. The inclusion of ApoE4 in multi-biomarker panels in clinical practice remains questionable due to regulatory restrictions, such as those imposed by the Genetic Diagnostics Act, which may limit its widespread use.

NfL was found to be the most reliable biomarker for CBS-Aβ(–), reflecting its role as a marker of axonal damage and brain atrophy in neurodegenerative diseases, underlining its usefulness in identifying non-amyloid pathologies. These findings were consistent with previous studies reporting elevated NfL levels in frontotemporal dementia, amyotrophic lateral sclerosis and other tauopathies [14–16]. The challenge with NfL lied in its limited sensitivity, as its diagnostic value for CBS-Aβ(–) relies on extreme elevations, highlighting the need for complementary markers to improve differentiation.

The limited contribution of GFAP to group differentiation was notable given its recognized role in AD as a marker of astroglial activation and early neuroinflammation, processes common to many neurodegenerative diseases [44, 45]. GFAP and pTau181 were grouped together by factor analysis, which provided an objective perspective. However, the overlaps of GFAP levels across the groups reduced its discriminatory power. While GFAP may be more relevant in preclinical stages or combined with other markers, its utility in this study was limited [44–48]. Based on the data, GFAP may serve more as a marker of general neurodegenerative processes rather than as a specific biomarker for distinguishing between diagnostic groups.

### Strengths of the study

This study has several strengths, including biomarker-guided participant selection, a comprehensive diagnostic work-up, and standardized procedures for sample handling and analysis. The uniform protocols minimize inconsistencies in sample handling and assay conditions, increasing the validity of the results. Automated assays have demonstrated high accuracy in detecting brain amyloid compared to amyloid PET, further enhancing reliability [3, 5, 49, 50].

### Limitations of the study

The small sample size, especially for CBS-Aβ(+) cases, limited the generalizability of the results and may have inflated AUC values, especially with multiple biomarkers. Smaller group sizes are likely to have increased the variance of AUC estimates, risking overfitting and overestimation of model performance. In addition, the clear clinical definitions in this highly selected population contributed to the high AUC values but limit the applicability of these findings to more heterogeneous cohorts. The Bayesian regression approach, including posterior distributions, provided robust uncertainty quantification to address potential overestimation of biomarker performance. Nevertheless, validation in larger, unselected cohorts will be essential to confirm these findings and refine biomarker applications in real-world settings.

Another limitation relates to the use of the MMSE as the primary cognitive assessment tool. Although it allows for standardized comparisons, its limited scope, particularly for assessing non-memory cognitive domains, reduces the granularity of subgroup differentiation. This is particularly relevant for CBS cases, as CBS often presents with heterogeneous cognitive and motor phenotypes that may be better captured by more detailed neuropsychological assessments [51]. Furthermore, grouping MCI due to AD and probable AD dementia into a single "Alzheimer's disease" group further limits stratification by disease stage, although it aligns with the continuum of disease progression observed in clinical practice [9, 22].

### Diagnostic utility of biomarkers

However, when considering diagnostic utility, the larger variability observed in BBBMs might be attributed to peripheral influences, such as inflammation or metabolic factors [39]. While CSF biomarkers are more directly linked to central nervous system pathology, BBBMs offer comparable diagnostic potential and greater accessibility.

## Conclusions

However, in this highly selected cohort, BBBMs showed remarkable potential to approach the discriminatory power of CSF and PET-based diagnostics, particularly in distinguishing AD from HC. This analysis highlights the strengths and limitations of BBBMs, demonstrating that while pTau181 and ApoE4 performed exceptionally well in discriminating AD from HC, the Aβ1-42/1-40 ratio exhibited some limitations and NfL emerged as a key marker in classifying CBS-Aβ(–), despite issues with sensitivity. These findings highlight the potential for BBBMs to serve as non-invasive alternatives to more invasive diagnostic modalities. The results emphasize tailored diagnostic strategies using multi-biomarker panels for specific group comparisons and the need for research to refine models and address confounding factors. Moving forward, validation in larger cohorts and unselected populations will be essential for the adoption of BBBMs to improve their reliability and applicability in different clinical contexts [52–54].

## Supplementary Information

Below is the link to the electronic supplementary material.

Supplementary file1 (XLSX 12 KB)

## Data Availability

The data supporting the findings of this study are available on request from the corresponding author. The data are not publicly available due to privacy or ethical restrictions.

## Abbreviations

AD: Alzheimer´s disease
Aβ: Amyloid-β
ApoE4: Apolipoprotein E protein (associated with lipid metabolism and AD risk)
ApoEε4: *APOE* Genotype variant (ε4 allele of the *APOE* gene)
BBBM: Blood-based biomarker
CERAD: The extended Consortium to Establish a Registry for Alzheimer's Disease battery
CBS: Corticobasal syndrome
CBS-Aß(+): Aß positive CBS cases
CBS-Aß(–): Aß negative CBS cases
CDR: Clinical Dementia Rating Scale
CSF: Cerebrospinal fluid
FCSRT: Free and Cued Selective Reminding Test
FDR: False Discovery Rate
GFAP: Glial fibrillary acidic protein
HC: Healthy control
MMSE: Mini Mental Status Examination
MCI: Mild cognitive impairment
n.a.: not applicable
NfL: Neurofilament light chain
p-tau181: Phosphorylated tau at threonine 181
PET: Positron emission tomography
TMT: Trail Making Test
SD: Standard deviation
SUVR: Standardized uptake value ratio
